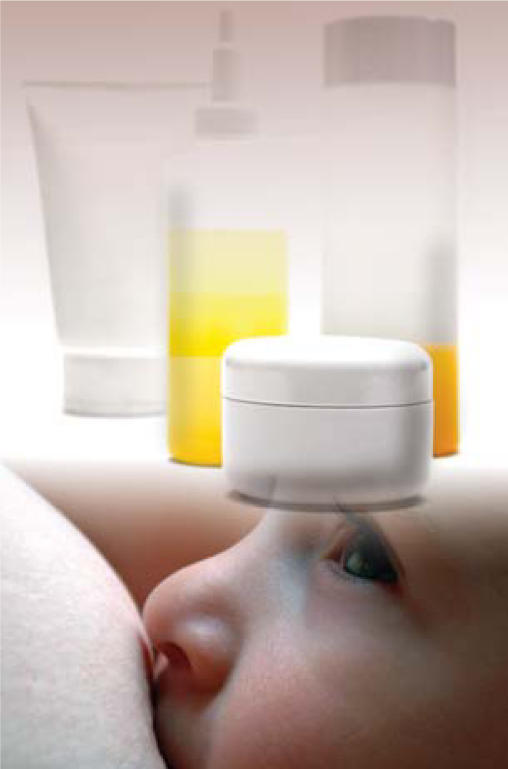# Chemical Exposures: The Sweet Scent on Baby’s Breath?

**DOI:** 10.1289/ehp.115-a491a

**Published:** 2007-10

**Authors:** Carol Potera

Synthetic fragrances known as polycyclic musks are added to soap, shampoo, deodorant, cleaning agents, cosmetics, and other consumer products. Now they are also turning up in human breast milk. In the first U.S. study to measure polycyclic musks in breast milk, environmental toxicologist Kurunthachalam Kannan at the New York State Department of Health and colleagues found the highest levels ever recorded in nursing mothers.

Over the past 20 years, polycyclic and other synthetic musks have replaced expensive natural musks derived from endangered wildlife. The two most widely used polycyclic musks, HHCB and AHTN, make up 90% or more of the U.S. and European markets for these compounds. Synthetic musks are used to mask chemical odors in products labeled “unscented”—though they aren’t added to products labeled “fragrance-free.”

Kannan measured polycyclic musks in milk samples collected from 39 nursing women in Massachusetts. The average concentration of HHCB was five times higher than that measured in European breast milk samples 10 years ago, and the average AHTN concentration was twice that detected in another European study in 1999. Using these averages and the average consumption of breast milk, Kannan estimated that babies may ingest 1,830 ng of HHCB and 5656 ng of AHTN per day.

Kannan believes pregnant women should avoid products with musk fragrances because, he says, “Not much is known about the toxicity of these compounds to humans, let alone to babies.” The findings are reported in the 1 June 2007 issue of *Environmental Science & Technology*.

Polycyclic musks are emerging pollutants, and little is known about their human or environmental health effects. Musk compounds in products like lotions can readily penetrate the skin. Inhaling airborne polycyclic musks (for example, from perfumes and air fresheners) offers another source of exposure. In an earlier study published in the November 2005 issue of *Chemosphere*, Kannan showed that polycyclic musks accumulate in human fat tissue at concentrations up to 1 ppm. Moreover, in the January 2005 issue of *EHP*, Till Luckenbach and David Epel showed that polycyclic musks blocked the activity of multidrug efflux transporters that pump toxicants from cells to protect them from damage. Inhibiting these pumps allows pollutants to enter cells and accumulate.

Manufacturers do not disclose the quantity of musk compounds added to consumer products yearly. Musk ingredients often are not listed on product labels, or they are called simply “fragrance” or by the trade names galaxolide (for HHCB) or tonalide (for AHTN). “Our everyday experience suggests that there are more fragrances in consumer products and at higher concentrations than ever before,” says Keri Hornbuckle, a professor of environmental engineering at the University of Iowa.

Hornbuckle’s group has measured polycyclic musks in surface water and outdoor air samples in rural and urban areas. “We find them everywhere,” she says, “and their concentration is proportional to the population of the area where samples are collected.” Concentrations of HHCB in Lake Erie doubled between 1990 and 1998, Hornbuckle reported in the 15 September 2006 issue of *Environmental Science & Technology*.

Hornbuckle also reported in the November 2006 *Archives of Environmental Contamination and Toxicology* that polycyclic musks reduce the growth of the juvenile and larval stages of the freshwater mussel *Lampsilis cardium*, a sentinel for vulnerable aquatic species in ecological systems. As scientists learn more about the impact of polycyclic musks on organisms and ecosystems, “we may become more concerned about exposure in humans,” Hornbuckle says.

## Figures and Tables

**Figure:**